# Comorbidities and Malignancy among NAFLD Patients Compared to the General Population, A Nation-Based Study

**DOI:** 10.3390/biomedicines11041110

**Published:** 2023-04-06

**Authors:** Naim Abu-Freha, Bracha Cohen, Michal Gordon, Sarah Weissmann, Alexander Fich, Daniela Munteanu, David Yardeni, Ohad Etzion

**Affiliations:** 1 The Institute of Gastroenterology and Hepatology, Soroka University Medical Center, Beer-Sheva 84101, Israel; 2 The Faculty of Health Sciences, Ben-Gurion University of the Negev, Beer-Sheva 84105, Israel; 3 Soroka Clinical Research Center, Soroka University Medical Center, Beer-Sheva 84101, Israel

**Keywords:** fatty liver, Israel, mortality, comorbidities, malignancy

## Abstract

(1) Background: Non-alcoholic fatty liver disease (NAFLD) is a common liver disease. Aims: We aimed to investigate the frequency of comorbidities and malignancies among NAFLD patients compared to the general population. (2) Methods: A retrospective study included adult patients with a NAFLD diagnosis. A control group was matched for age and gender. Demographics, comorbidities, malignancies, and mortality were collected and compared. (3) Results: 211,955 NAFLD patients were analyzed in comparison to 452,012 matched general population controls. Significantly higher rates of diabetes mellitus (23.2% vs. 13.3%), obesity (58.8% vs. 27.8%), hypertension (57.2% vs. 39.9%), chronic ischemic heart disease (24.7% vs. 17.3%), and CVA (3.2% vs. 2.8%) were found among NAFLD patients. Patients with NAFLD had significantly higher rates of the following malignancies: prostate cancer (1.6% vs. 1.2%), breast cancer (2.6% vs. 1.9%), colorectal cancer (1.8% vs. 1.4%), uterine cancer (0.4 vs. 0.2%), kidney cancer (0.8% vs. 0.5%), but a lower rate of lung cancer (0.9% vs. 1.2%) and stomach cancer (0.3% vs. 0.4%). The all-cause mortality rate among NAFLD patients was significantly lower in comparison to the general population (10.8% vs. 14.7%, *p* < 0.001). (4) Conclusions: Higher rates of comorbidities and malignancies among NAFLD patients were observed, but a lower rate of all-cause mortality was found.

## 1. Introduction

Non-alcoholic fatty liver disease (NAFLD) is a common and emerging liver disease that is considered to be the major cause of chronic liver disease, with a global prevalence of approximately 25% of the adult population in the western world and varying prevalence between different regions worldwide [1,2]. In general, NAFLD is considered the hepatic manifestation of the metabolic syndrome and is associated with insulin resistance. Due to the high prevalence of NAFLD, the proportion of liver-related complications resulting from NAFLD, such as cirrhosis, hepatocellular carcinoma (HCC), liver transplantation, and mortality, is expected to be higher than for other aetiologies and to have an enormous clinical and economic burden [1].

Different medical comorbidities such as diabetes mellitus, obesity, dyslipidemia, hypertension, cardiovascular disease, endocrine diseases (hypothyroidism, osteoporosis), and cancers are prevalent among NAFLD patients [3,4,5], with varying reported prevalence [1,2,3,4,5]. Obesity prevalence is estimated to be about 51% of NAFLD patients and about 82% of nonalcoholic steatohepatitis (NASH) patients, while the prevalence of diabetes, hypertension, and dyslipidemia are 22%, 40%, and 69% among NAFLD patients, with an even higher prevalence in NASH patients [1]. Other studies showed an increased risk of non-fatal cardiovascular events with an odds ratio of 2.52 in NAFLD patients compared to non-NAFLD patients, as well as an increased risk of congestive heart failure, atrial fibrillation, ischemic stroke, and chronic kidney disease [6,7,8,9]. However, there are only scant data published regarding the comparison of NAFLD patients and the general population. The most common causes of death among NAFLD patients are cardiovascular diseases, followed by extrahepatic malignancies [10,11,12]. In general, there has been an increase in the incidence and prevalence of malignancy among NAFLD patients [10], and liver cancer [13]. Allen et al. followed 4722 patients with NAFLD and 14,441 controls and found an increased risk of cancer among NAFLD patients with an incidence rate ratio (IRR) of 1.9, in particular gastrointestinal cancers [10]. The IRRs of stomach, pancreas, and colon cancer were 2.3, 2.0, and 1.8, respectively. In the absence of NAFLD, the association between obesity and cancer risk was small, suggesting that NAFLD is the main factor for increased cancer risk [10].

We aimed to determine the frequency of important comorbidities and malignancies among NAFLD patients and their timeline in comparison to the general population as a control group.

## 2. Materials and Methods

### 2.1. Population and Study Setting

Patients aged 18 years and older diagnosed with NAFLD (extracted from the diagnosis list in the community clinic or hospital) were enrolled in the analysis. Patients were included in the study if they had a diagnosis of NAFLD or non-alcoholic steatohepatitis in a community clinic, during a hospitalization, or on a list of chronic problems. Patients with diagnoses other than liver diseases, such as alcohol abuse or hepatitis B or C, were excluded from the study group but not from the control group. The data were extracted from Clalit Health Services (CHS) using Clalit’s data sharing platform powered by MDClone (https://www.mdclone.com, accessed on 1 March 2023). CHS is the largest health maintenance organization (HMO) in Israel, with about 4.7 million insured residents, accounting for 53% of the Israeli population. The study subjects with NAFLD were compared to the control group. Control group participants were taken from the general population insured by Clalit HMO and were matched with NAFLD patients according to age and gender.

### 2.2. Data Collection

Demographics, comorbidities, malignancies, complications, outcomes, and mortality of NAFLD patients and controls were retrospectively collected according to the ICD codes for diagnosis. Data regarding the metabolic syndrome (diabetes mellitus, obesity, dyslipidemia, and hypertension), cardiovascular diseases (chronic ischemic heart disease, cerebrovascular accidents), lung diseases, and renal failure were collected. In addition, the frequency of common malignancies was collected (lung, prostate, breast, colorectal, stomach, pancreatic, uterine, and ovarian cancers).

Outcomes included cirrhosis, hepatocellular carcinoma, liver transplantation, and mortality. All of the mentioned data were compared between NAFLD patients and the control group. All data regarding the comorbidities, malignancies, and outcomes were collected according to the ICD-10 diagnosis in the medical files.

### 2.3. Statistical Analysis

Data are presented as means ± standard deviations (SD) for continuous variables and as percentages (%) of the total for categorical variables. Univariate analyses were performed by the Mann–Whitney test (for continuous variables), Fisher’s exact, and chi-squared tests (for categorical variables). All statistical analyses were performed using IBM SPSS version 26 (Chicago, IL, USA).

*p*-Values less than 0.05 were considered statistically significant. The study was carried out in accordance with the principles of the Helsinki Declaration. The study protocol was approved by the Institutional Helsinki Committee, approval number 198-21-SOR.

## 3. Results

### 3.1. This Study Population

A total of 211,955 patients with NAFLD were included in the analysis, with a mean age at diagnosis of 42.2 ± 15 years and a 47.2% male composition. The matched control group included 452,012 subjects with a mean age of 42.4 ± 14.8 and a 48.5% male composition. The BMI of NAFLD patients was significantly higher than the control group (29.78 ± 6 compared to 26.98 ± 5.8, *p* < 0.001). The follow-up time was 19.89 ± 4.1 years for the NAFLD cases and 19.95 ± 4 years for the controls.

### 3.2. Comorbidities

The frequency of comorbidities in the NAFLD group and the control group is presented in Table 1. Among patients with NAFLD, we found higher frequencies of all metabolic syndrome-related diseases compared to the general population: diabetes mellitus (23.2% vs. 13.3%, *p* < 0.001), obesity (58.8% vs. 27.8%, *p* < 0.001), dyslipidemia (69.5% vs. 47%, *p* < 0.001), and hypertension (57.2% vs. 39.9%, *p* < 0.001). In addition, higher rates of vascular diseases, including chronic ischemic heart disease (24.7% vs. 17.3%, *p* < 0.001) and cerebrovascular accidents (3.2% vs. 2.8%, *p* < 0.001) were found. Higher rates of other diseases, such as lung chronic disease, chronic renal failure, dementia, vitamin B12 deficiency, folic acid deficiency, and iron deficiency, were also found among NAFLD patients.

### 3.3. Malignancy

Extrahepatic malignancy was diagnosed in 116,580 (17.6%) patients.

During the follow-up period, 43,501 (20.5%) NAFLD patients were diagnosed with cancer, while 73,079 (16.2%) control subjects were diagnosed with cancer (*p* < 0.001). Malignancy diagnosis rates among the NAFLD population and the control group are summarized in Table 2.

During the follow-up period, higher rates of prostate cancer, colorectal cancer, breast cancer, uterine cancer, kidney cancer, melanoma, basal cell carcinoma, thyroid carcinoma, and non-Hodgkin’s lymphoma were diagnosed among NAFLD patients, while significantly lower rates of lung cancer, stomach cancer, and lymphoma were diagnosed among NAFLD patients. There was no significant difference between NAFLD patients and the general population regarding the frequency of pancreatic cancer or ovarian malignancy.

### 3.4. Timeline of Comorbidities Diagnosed in NAFLD Patients

**Figure 1:**
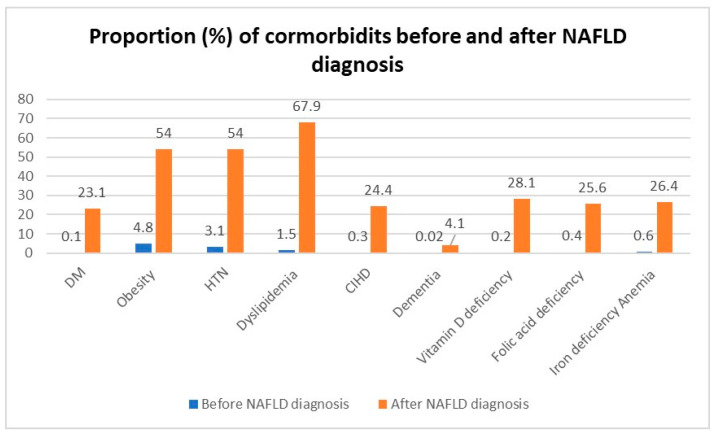
Timeline of comorbidities diagnosis compared to NAFLD.

The timeline of comorbidities and the percentage of patients diagnosed with comorbidities before and after their NAFLD diagnosis are shown in Figure 1. We found that most comorbidities, including metabolic syndrome diseases, were diagnosed after the diagnosis of NAFLD. For example, 23.1% of NAFLD patients were diagnosed with diabetes mellitus after their NAFLD diagnosis, while only 0.1% of them were diagnosed before their NAFLD diagnosis. Similarly, 54% of NAFLD patients were diagnosed with obesity after NAFLD diagnosis, compared with 4.8% of patients who were diagnosed with obesity before NAFLD diagnosis. In addition, deficiencies such as vitamin B12, vitamin D, and iron deficiency anemia were diagnosed after the diagnosis of NAFLD.

### 3.5. Timeline of Malignancy Diagnoses in NAFLD Patients

Only a very small proportion of the malignancy diagnoses were made before NAFLD diagnoses. Most malignancies were diagnosed after NAFLD. A total of 1996 cases of lung cancer were diagnosed after the diagnosis of NAFLD while 5 cases were diagnosed before NAFLD, 3450 cases of prostate cancer were diagnosed after NAFLD diagnosis while 40 cases were diagnosed before, 3714 cases of colorectal cancer were diagnosed after the diagnosis of NAFLD while only 19 cases were diagnosed before NAFLD, and 5538 cases of breast cancer were diagnosed after NAFLD diagnosis while only 11 cases were diagnosed before NAFLD.

### 3.6. Complications and Mortality

Liver-related complications and all-cause mortality rates are summarized in Table 3. Cirrhosis was diagnosed in 2.1% of the NAFLD population compared to 0.9% of the general population (*p* < 0.001). Cirrhosis was diagnosed at an older age among NAFLD patients compared to the general population (64.86 ± 13 years vs. 61.54 ± 12.3 years, *p* < 0.001) and all cirrhosis-related complications (HCC, SBP, HRS, and esophageal varices) were more common among NAFLD patients compared to the general population. In addition, higher rates of liver transplantation were found among NAFLD patients compared to the general population (0.1% vs. 0.06%, *p* < 0.001). The all-cause mortality rate was lower among NAFLD patients than the general population, 10.8% vs. 14.7%, *p* < 0.001, and the age of death was older among NAFLD patients, 76.12 ± 12.3 years vs. 71.4 ± 12.9 years, *p* < 0.001.

## 4. Discussion

Understanding NAFLD in terms of comorbidities and malignancies is crucial for planning future interventions and disease prevention. In the present study, we investigated the common comorbidities and malignancies among NAFLD patients compared to the general population. In order to reach our aim in this study, we included a large number of NAFLD patients (211,955 patients) and compared them to a large number of control subjects (452,012 individuals).

The most important findings our study showed were higher rates of comorbidities, malignancies, and liver-related complications among NAFLD patients compared to the general population, but a lower rate of all-cause mortality than the general population.

In our cohort, we found significantly higher rates of comorbidities, not only metabolic syndrome-related but also other non-metabolic-related comorbidities. We found 23.2% of NAFLD patients had diabetes mellitus compared to 13.3% of the general population, and 58.5% of NAFLD patients were obese compared to 27.8% of the general population. In addition, metabolic syndrome-related vascular diseases such as cardiac vascular disease and cerebrovascular accidents were more common among NAFLD patients. Moreover, significantly higher rates of chronic lung disease, chronic renal failure, vitamin B12 deficiency, folic acid deficiency, and iron deficiency anemia were found among NAFLD patients compared to the general population. When comparing our results with those in a previous large meta-analysis, our cohort had higher rates of obesity and hypertension (58.5% vs. 51.34% and 57.2% vs. 39.34%, respectively), but similar rates of diabetes mellitus type 2 and hyperlipidemia [1]. According to a different study, higher rates of diabetes mellitus and chronic renal disease were reported compared to our findings [14], which could indicate that the prevalence of comorbidities among NAFLD patients may differ from one region to another but, as we observed, is still significantly higher than the general population.

The relationship between NAFLD and other comorbidities is complex, with related factors including the prevalence of the comorbidity, the timeline of the diagnosis, and the pathophysiological effects. Changes in lifestyle and dietary patterns due to NAFLD and other comorbidities influence the prevalence of diseases related to diet, particularly obesity. Rates of obesity are also increasing worldwide, and the WHO report found 39% of all adults worldwide in 2014 were overweight, about a twofold increase from 1980 [15].

Another important aspect that has not been previously studied is the timeline of NAFLD diagnosis compared to other comorbidities. The mean age at the time of NAFLD diagnosis was 42.2 years. In our study, we found that most comorbidities were diagnosed after the diagnosis of NAFLD, especially comorbidities related to metabolic syndrome. Among NAFLD patients who have diabetes mellitus, only 0.1% were diagnosed before NAFLD, and 23.1% were diagnosed after NAFLD. 4.8% of NAFLD patients were diagnosed with obesity before NAFLD, while 54% were diagnosed after NAFLD. Figure 1 presents the proportion of comorbidities diagnosed before and after NAFLD.

The pathophysiological mechanism behind NAFLD is an important factor that could contribute to the development of comorbidities. The systemic inflammatory process, insulin resistance, change in vascular stiffness, increased oxidative stress, gut dysbiosis, and genetic and epigenetic modifications could play a role in the interaction between NAFLD and the other comorbidities (hypertension, obesity, and cardiovascular disease) [16]. Diet is an important factor in NAFLD development, as is the microbiome and its relationship with NAFLD. NAFLD and metabolic syndrome development are influenced by an individual’s dietary pattern; the western diet (rich in sugars, saturated fat, and red meat) is associated with liver fat deposition while the Mediterranean diet (vegetables, fruit, legumes, aromatic herbs, and extra virgin olive oil) is associated with improvement of intrahepatic fat accumulation [17,18,19,20,21,22,23].

In the last few years, increased interest and knowledge regarding the pathophysiology of NAFLD, the role of the microbiome, and NAFLD-associated diseases such as diabetes mellitus have come to light. Multiple factors (environmental, genetic, and comorbidities) have been shown to modify the gut microbiota [24,25,26]. Other studies have demonstrated a correlation between a specific microbial signature and the severity of NAFLD and the prediction of NAFLD-related cirrhosis [27,28,29].

Malignancies among NAFLD patients were the second field we focused on in this study, and we found that the prevalence of the malignancy is higher among NAFLD patients than the general population (20.5% of NAFLD patients compared to 16.2% of the general population). Most types of malignancies were more common among NAFLD patients than the general population (melanoma, prostate, colorectal, breast, uterine, and kidney cancer), except lung and stomach cancer, which were found less commonly among NAFLD patients compared to the general population. There was no significant difference between NAFLD patients and the general population with regard to pancreatic and ovarian cancer. The malignancy rates among both groups in our study were higher than in other previous reports; 15.3% of NAFLD patients and 13.4% of the general population were diagnosed with a malignancy in one previous study [30]. Another study showed 16.5% of NAFLD patients and 12.2% of controls were diagnosed with cancer [14]. Previous study showed an increased prevalence of specific cancers, such as gastrointestinal tract, liver, and uterine cancers [10], and one study showed increased rates of male genital cancer (including the penis, prostate, and testicular cancers), skin cancer, including malignant cutaneous melanoma, and breast cancer in NAFLD patients [30].

Both the prevalence of malignancy worldwide and the rate of malignancy among NAFLD patients are increasing. However, we still do not have enough of an understanding of the factors that contribute to the increased risk of malignancy among NAFLD patients. Several factors have been suggested, such as metabolic inflammation with complex inflammatory cascades, which include an effect on cellular dysfunction as well as cell death and the secretion of a large number of different mediators. In addition, several comorbidities that are common among NAFLD patients, such as diabetes mellitus and obesity, are known to be risk factors for malignancy [31,32]. There is no doubt that more in-depth research is necessary to improve our understanding of specific risk factors for cancer among NAFLD patients, including not only comorbidities but also inflammatory and carcinogenic processes and the relationship between both and malignancy in this population.

The incidence of both NAFLD and malignancy, irrespective of NAFLD, is increasing, so it is expected that malignancy rates among NAFLD will continue to increase. Until we understand the development of malignancy among NAFLD patients, special interventions are needed in order to treat and interrupt the inflammatory process of NAFLD. NAFLD patients should be considered high-risk patients for malignancy, and improvements in adherence to screening and diagnosis of malignancy in early stages are critical, particularly because most malignancies are diagnosed after the diagnosis of NAFLD.

In this study, we found a lower all-cause mortality rate among NAFLD patients but a higher rate of liver complications (cirrhosis, hepatocellular carcinoma, esophageal varices, spontaneous bacterial peritonitis, and hepatorenal syndrome) than the general population. The NAFLD group included patients with chronic liver disease, whereas only a small proportion of the control group had chronic liver disease, so it is expected that these patients will develop more liver-related complications. It is important to mention that despite the higher rate of liver complications and higher rates of most comorbidities and malignancies, the all-cause mortality rate was significantly lower among the NAFLD patients, at 10.8% compared to 14.7% among the general population. In general, because of the higher rate of comorbidities and malignancy rates among NAFLD patients, a higher rate of all-cause mortality would be expected among the NAFLD patients, and this has been confirmed in previous study [33]. Though there are no clear explanations for our finding, some explanations could include: (1) increased follow-up with higher awareness for symptoms and general health due to a NAFLD diagnosis, leading to early diagnosis and treatment of comorbidities and malignancy; (2) lifestyle changes due to a NAFLD diagnosis that subsequently improve the burden of comorbidities and therefore morbidity and mortality; and (3) a relatively young mean age at diagnosis of NAFLD (42 years) in our cohort that may contribute to the lower all-cause mortality. Increased awareness of symptoms and general health may not only decrease all-cause mortality via early diagnosis and treatment of comorbidities and malignancies, but it may also be seen in the increased diagnoses of comorbidities following NAFLD diagnosis. A recently published study with a median follow-up of 22.8 years showed that a healthy lifestyle reduced all-cause mortality by 36% and cardiovascular disease-related mortality by 43%, possibly explaining the decrease in all-cause mortality in our population [34]. Nevertheless, the effect of NAFLD on all-cause mortality remains controversial, with most of the studies showing increased mortality risk and others showing no increased risk for all-cause mortality [35,36,37,38,39].

To summarize, in comparison to the general population, NAFLD patients have a higher risk of developing a variety of comorbidities, not only metabolic-related diseases. In addition, NAFLD patients have a higher risk of most types of malignancy than their healthy counterparts. NAFLD patients are diagnosed at a young age, and most comorbidities and malignancies are diagnosed later. There is still a lack of understanding as to whether these comorbidities cause increased NAFLD or whether NAFLD causes these comorbidities. In our study, we found that NAFLD was diagnosed before most comorbidities, which strengthens the evidence that the relationship between NAFLD and comorbidities is bidirectional. It is possible that NAFLD is not only a consequence of these comorbidities but also appears early in the metabolic syndrome, increasing the risk of other related and unrelated comorbidities. Our findings regarding the timeline of NAFLD diagnoses compared to other comorbidities should be confirmed in future studies in different regions. Nevertheless, this is an important finding that highlights the importance of education and awareness of comorbidities and malignancies and points out the need for prevention and intensive treatment in these patients. The importance of our finding lies in increasing knowledge regarding NAFLD comorbidities, related malignancies, and their timeline in relation to NAFLD. These are essential for the early detection of comorbidities and malignancies and the treatment and follow-up of these diseases.

The strengths of our study are the large number of NAFLD patients and the use of matched controls, as well as the inclusion of more comorbidities and malignancies than in other previous studies. However, there are several limitations study that are important to mention. First, the retrospective design of our study used diagnoses based on ICD-10 codes, with possible misclassification or incorrect coding or potentially missed diagnoses due to asymptomatic disease presentation. Second, our study results include all-cause mortality and not just liver-specific mortality. Third, no data regarding smoking’s socioeconomic status, diet pattern, or physical activity were available.

## 5. Conclusions

In conclusion, NAFLD is a systemic disease with higher rates of comorbidities (metabolic syndrome-related and non-related) and malignancies compared to the general population. NAFLD was diagnosed earlier than most other comorbidities and malignancies, but the all-cause mortality rate of NAFLD patients was still lower than that of the general population.

## Figures and Tables

**Table 1:** Baseline characteristics and comorbidities of NAFLD and non-NAFLD groups.

Variable	NAFLD 211,955 (%)	Non-NAFLD Controls 452,012 (%)	*p*-Value
Sex, male	99,962 (47.2)	219,267 (48.5)	<0.001
Age at diagnosis, mean ± SD	42.2 ± 15	42.4 ± 14.8	<0.001
Age, mean ± SD	62.3 ± 15.8	62 ± 15.1	<0.001
Ethnicity, Arabs	34,090 (16)	74,345 (16.4)	<0.001
Comorbidities			
Diabetes Mellitus	49,263 (23.2)	60,253 (13.3)	<0.001
Obesity	124,577 (58.8)	125,638 (27.8)	<0.001
Dyslipidemia	147,354 (69.5)	212,369 (47)	<0.001
Hypertension	121,202 (57.2)	180,305 (39.9)	<0.001
Cardiovascular disease	52,300 (24.7)	78,043 (17.3)	<0.001
CVA	6723 (3.2)	12,649 (2.8)	<0.001
COPD	21,456 (10.1)	37,540 (8.3)	<0.001
Asthma	30,221 (14.3)	43,492 (9.6)	<0.001
CRF	27,333 (12.9)	42,914 (9.5)	<0.001
Dementia	8702 (4.1)	17,589 (3.9)	<0.001
Vit B12 deficiency	2712 (1.3)	4324 (1)	<0.001
Folic acid deficiency	54,968 (25.9)	79,454 (17.6)	<0.001
Iron deficiency anemia	57,183 (27)	83,696 (18.5)	<0.001
Hepatitis C	0	6246 (1.4)	<0.001
Hepatitis B	0	6188 (1.4)	<0.001
Alcohol Abuse	0	9832 (2.17)	<0.001

CVA—Cerebrovascular Accident; COPD—Chronic Obstructive Pulmonary Disease; CRF—Chronic Renal Failure.

**Table 2:** Malignancy rates among NAFLD and Non-NAFLD controls.

Variable	NAFLD 211,955 (%)	Non-NAFLD Controls 452,012 (%)	*p*-Value
Any cancer	43,501 (20.5)	73,079 (16.2)	<0.001
Prostate (% of men)	3490 (3.5)	5506 (2.5)	<0.001
Colorectal Cancer	3733 (1.8)	6193 (1.4)	<0.001
Breast	5549 (2.6)	8687 (1.9)	<0.001
Uterus (% of women)	901 (0.8)	1095 (0.5)	<0.001
Kidney	1598 (0.8)	2113 (0.5)	<0.001
Melanoma	3457 (1.6)	5404 (1.2)	<0.001
Basal cell carcinoma	24,649 (11.6)	38,918 (8.6)	<0.001
Thyroid carcinoma	1543 (0.7)	2053 (0.5)	<0.001
Non-Hodgkin lymphoma	1548 (0.7)	2552 (0.6)	<0.001
Lung cancer	2001 (0.9)	5637 (1.2)	<0.001
Stomach	663 (0.3)	1590 (0.4)	0.011
Lymphoma	494 (0.2)	744 (0.2)	<0.001
Pancreas	551 (0.3)	1145 (0.3)	0.617
Ovarian (% of women)	694 (0.6)	1376 (0.6)	0.310

**Table 3:** Complications and mortality among NAFLD and non-NAFLD patients.

Variable	NAFLD 211,955	Non-NAFLD Controls 452,012	*p*-Value
Cirrhosis	4429 (2.1)	4190 (0.9)	<0.001
Age at cirrhosis, mean ± SD	64.8 ± 13	61.5 ± 12.3	<0.001
HCC	943 (0.44)	1586 (0.35)	<0.001
Age of HCC	68.8 ± 11.9	67.1 ± 10.7	<0.001
Esophageal varices	1278 (0.6)	1136 (0.3)	<0.001
Esophageal varices bleeding	647 (0.3)	678 (0.1)	<0.001
SBP	303 (0.14)	314 (0.07)	<0.001
Hepatorenal syndrome	284 (0.12)	276 (0.06)	<0.001
Liver transplantation	194 (0.1)	272 (0.06)	<0.001
Age at liver t transplantation, mean ± SD	55 ± 11.9	55.5 ± 10	0.656
Death	22,963 (10.8)	66,418 (14.7)	<0.001
Age at death, mean ± SD	76.1 ± 12.3	71.4 ± 12.9	<0.001
Number of hospitalizations From diagnosis, mean ± SD	4.3 ± 6.5	3.1 ± 5.6	<0.001

HCC—Hepatocellular Carcinoma; SBP—Spontaneous Bacterial Peritonitis.

## Data Availability

No additional data are available.
